# Utility of *Ochrobactrum anthropi* YC152 in a Microbial Fuel Cell as an Early Warning Device for Hexavalent Chromium Determination

**DOI:** 10.3390/s16081272

**Published:** 2016-08-16

**Authors:** Guey-Horng Wang, Chiu-Yu Cheng, Man-Hai Liu, Tzu-Yu Chen, Min-Chi Hsieh, Ying-Chien Chung

**Affiliations:** 1 Fujian Provincial Key Laboratory of Biological Engineering on Traditional Herbs and Research Center of Natural Cosmeceuticals Engineering, Xiamen Medical College, Xiamen 361008, China; wanggh@livemail.tw; 2 Department of Biological Science and Technology, China University of Science and Technology, Taipei 11581, Taiwan; cycheng@cc.cust.edu.tw (C.-Y.C.); HBO223080284@gmail.com (T.-Y.C.); tsakaguchi22@gmail.com (M.-C.H.); 3 Department of Food Science, China University of Science and Technology, Taipei 11581, Taiwan; manhailiu@cc.cust.edu.tw

**Keywords:** chromiun, biosensor, microbial fuel cell, wastewater

## Abstract

Fast hexavalent chromium (Cr(VI)) determination is important for environmental risk and health-related considerations. We used a microbial fuel cell-based biosensor inoculated with a facultatively anaerobic, Cr(VI)-reducing, and exoelectrogenic *Ochrobactrum anthropi* YC152 to determine the Cr(VI) concentration in water. The results indicated that *O. anthropi* YC152 exhibited high adaptability to pH, temperature, salinity, and water quality under anaerobic conditions. The stable performance of the microbial fuel cell (MFC)-based biosensor indicated its potential as a reliable biosensor system. The MFC voltage decreased as the Cr(VI) concentration in the MFC increased. Two satisfactory linear relationships were observed between the Cr(VI) concentration and voltage output for various Cr(VI) concentration ranges (0.0125–0.3 mg/L and 0.3–5 mg/L). The MFC biosensor is a simple device that can accurately measure Cr(VI) concentrations in drinking water, groundwater, and electroplating wastewater in 45 min with low deviations (<10%). The use of the biosensor can help in preventing the violation of effluent regulations and the maximum allowable concentration of Cr(VI) in water. Thus, the developed MFC biosensor has potential as an early warning detection device for Cr(VI) determination even if *O. anthropi* YC152 is a possible opportunistic pathogen.

## 1. Introduction

Various industrial activities, such as steel production, leather tanning, agro-food production, wood preservation, and chemical manufacturing, generate wastewater containing Cr(VI) [1]. Chromium generally exists in water in the following two oxidation forms: hexavalent [Cr(VI)] and trivalent [Cr(III)]. The Cr(VI) concentration above a specific dose is toxic, mutagenic, and carcinogenic [2,3]. Compared with Cr(VI), Cr(III) is less toxic and can be more readily precipitated out of the solution in the form of Cr(OH)_3_; it is also impermeable to biological membranes [4]. By contrast, Cr(VI) is highly soluble and, thus, mobile and biologically available in ecosystems. Thus, authorities worldwide have stringent regulations for chromium species concentrations, especially Cr(VI). The U.S, Environmental Protection Agency identified Cr(VI) as one of the 17 chemicals posing the greatest threat to humans [5]. In Taiwan, the standard concentration of industrial and domestic effluents for Cr(VI) is 0.5 mg/L, and the maximum allowable concentration of Cr(VI) in drinking and surface water is more stringent, at 0.05 mg/L.

Environmental cleanup technologies for Cr(VI) removal from wastewater involve chemical precipitation, chemical oxidation, ion exchange, electrochemical treatment, reverse osmosis, membrane technology, and biological detoxification [4]. These processes may be ineffective, especially when the metal concentration in the solution is below 100 mg/L [6]. Moreover, the accidental release of industrial effluents without proper treatment would pose a serious threat to the environment. Thus, the determination of chromium species concentrations, especially of Cr (VI), in water is crucial for environmental risk and health-related considerations.

The currently available and widely-used analytical methods and techniques for the determination of chromium ions include atomic absorption spectroscopy (AAS), inductively coupled plasma mass spectroscopy, ion chromatography, and their combination with chromatographic techniques [7]. Although these techniques exhibit high sensitivity, accuracy, and selectivity, they are usually tedious, time consuming, expensive, and complicated. Moreover, they often require sophisticated instrumentation inadequate for use outside the laboratory; thus, they have low applicability in routine determinations [8,9]. Compared with these traditional analytical methods and techniques, a simple, inexpensive, and portable biosensor is a more feasible option. If a biosensor provides rapid measurements with acceptable accuracy, it can become a potential early warning detection device that can be used to protect ecosystems [10].

A biosensor is generally defined as a self-contained integrated device capable of providing quantitative or semi-quantitative information by using a biological recognition element and that is also specific for the compound that you want to detect [11]. Several biosensor types have been developed, including DNA-based, whole cell-based, and enzyme-based biosensors, for determining the Cr(VI) concentration [12].

Michel et al. (2006) constructed a Cyt c3-based biosensor to measure the Cr(VI) concentration in groundwater [13]. However, the response of this biosensor is affected by pH, temperature, ionic strength, oxygen content, and sulfate concentration, and the detection limit for Cr(VI) is 0.2 mg/L. In addition, Nepomuscene et al. (2007) developed a urease-based biosensor for determining the Cr(VI) concentration in wastewater [14]. Although the operational stability and reproducibility of this biosensor are satisfactory, short urease storage periods and high detection limits for Cr(VI) restrict its commercial application. Gurung et al. (2012) constructed a cell-based biosensor by using sludge containing sulfur-oxidizing bacteria (SOB) to detect the Cr(VI) concentration on the basis of the metabolic properties of SOB and obtained preliminary results [15]. The tested concentration of this system for Cr(VI) is 1 mg/L, which is not suitable to meet the requirement of effluent regulations (<0.05 mg/L). Furthermore, Bohrn et al. (2013) constructed a cell-based biosensor by using V79 hamster lung fibroblast cells as the biological recognition element. The detection limit of this biosensor is 0.026 mg/L for Cr(VI) within 6 h of exposure [16]. Although the V79 cell-based biosensor is a powerful tool for detecting Cr(VI) concentrations in the range of multinational drinking water regulations, the high cost of this biosensor limits its applicability. Panda and Sarkar (2014) developed a stable biosensor by using crude cell-free extracts of *Enterobacter aerogenes* immobilized with calcium alginate beads for the direct estimation of Cr(VI) in wastewater [17]. This biosensor has an excellent limit of detection of 0.0066 mg/L for Cr(VI); however, it is extremely sensitive to higher Cr(VI) concentrations (e.g., >0.04 mg/L). Moreover, the concentration range for detecting Cr(VI) concentrations in water is, reportedly, very narrow.

Calvo-Pérez et al. (2014) constructed a novel enzyme-based biosensor for measuring the Cr(VI) concentration by using glucose oxidase as the biological element and a screen-printed carbon electrode as the transduction element [9]. This biosensor exhibited a linear range for Cr(VI) concentration of 0.006–0.048 mg/L, which is suitable for measuring trace Cr(VI) in tap and drinking water. Recently, a study reported that fluorescent cell-based biosensors including pCHRGFP1 *Escherichia coli* and pCHRGFP2 *Ochrobactrum tritici* are suitable for detecting the Cr(VI) concentration in environmental waters [18]. Coelho et al. (2015) reported that the pCHRGFP1 *E. coli* and pCHRGFP2 *O. tritici* biosensors functioned within the range of 0.031–0.124 mg/L and 0.124–0.620 mg/L, respectively, for detecting Cr(VI) concentration [19]. However, the fluorescence activity of the cryopreserved cells of the pCHRGFP2 *O. tritici* reporter might be up to 47% lower than the fluorescence activity of these cells in fresh reporters.

A microbial fuel cell (MFC) is a device using microorganisms as catalysts to generate electricity from chemical compounds. It has become commonly cited as a potential alternative for energy production because of its lower pollution levels, low cost, and wide applicability [20]. A typical MFC design consists of two compartments: one is anaerobic (i.e., the anode) and the other is aerobic (i.e., the cathode). Bacteria oxidize the substrate, generating electrons and protons in the anaerobic compartment. The electrons transfer to the anode either by the mediator, an exogenous electron carrier, or directly from the bacterial enzymes to the electrode. The protons transfer to the cathode compartment [21]. In addition, MFCs can produce a signal for practical applications such as powering electronic sensors to analyze pollutants and monitoring state variables for system control [22,23]. At present, MFC biosensors are applied to detect biochemical oxygen demand (BOD), toxicity, (volatile fatty acid) VFA, and Nickel (Ni) in wastewater and are validated to minimize the time and the cost [23,24,25,26]. Theoretically, most microbes can potentially be used as a biocatalyst in MFCs. However, anaerobic bacteria are often used in the anode compartment of MFCs because this compartment is designed under anaerobic conditions. Chromate reduction by bacteria occurs under aerobic or anaerobic conditions, and anaerobic reductions proceed through the use of Cr(VI) as a terminal electron acceptor [5]. Chromate-reducing bacteria include *Arthrobacter aurescens*, *Arthrobacter* sp., *Bacillus cereus S-6*, *B. subtilis*, *Micrococcus* sp. SDCr-4, *O. anthropi* CTS-325, *Oscillatoria* sp. BJ2, *Providencia* sp., *Streptomyces griseus*, and *Synechocystis* sp. and can remove Cr(VI); however, the strains nearly belong to aerobic strains [27], which are not used in MFCs. Other major players, like *Shewanella oneidensis* MR-1; a facultative anaerobe, Cr^6+^ reducer and exoelectrogen, has been used as a biocathode in MFCs to reduce Cr(VI) [28]. The possible mechanism for inoculating chromate-reducing bacteria in the anode compartment of MFC is as follows:

Anode: Organics → CO_2_ + H^+^ + e^−^ (by chromate-reducing bacteria)

Cr^6+^ + e^−^ → Cr^3+^ (by chromate-reducing bacteria)

Cathode: O_2_ + H^+^ + e^−^ → H_2_O (by chemical reaction)

The higher the Cr^6+^ concentration exists in the anode, the fewer electrons are transferred to the cathode if organic concentrations remain constant. Thus, the potential output will decrease with the increasing Cr^6+^ concentration.

In this study, *O. anthropi* YC152, a facultatively anaerobic, Cr(VI)-reducing, and exoelectrogenic bacterium, was isolated from wastewater containing Cr(VI). It was inoculated in an MFC to evaluate its feasibility as a biosensor or an early warning device for the detection of Cr(VI). Crucial operating parameters were established to optimize the performance of the MFC. The relationship between the voltage output and Cr(VI) concentration was investigated.

## 2. Materials and Methods

### 2.1. Bacterial Strains, Cultivation, and Identification

Sludge samples were collected from an electroplating wastewater treatment plant in Taoyuan City, Taiwan, and then centrifuged at 8000× g for 40 min. Precipitates were inoculated in a 3-L working volume of a chemostat and mixed with Luria Bertani (LB) broth supplemented with Na_2_Cr_2_O_7_, (LBCr medium). Subsequently, the LBCr medium containing 5–100 mg/L of Cr(VI) was progressively added into the chemostat to acclimate Cr(VI)-resistant or -reducing bacteria under the anaerobic conditions at 35 °C and a liquid retention time (LRT) of 24 h. A dominant strain (YC152) was isolated from the chemostat by using the spread plate method after a 36-d acclimation period. To identify the isolated YC152 bacterium, cell lysis, DNA extraction, 16S rRNA gene amplification, and sequencing were performed as described previously [29]. The genomic DNA of YC152 bacterium was extracted using a DNeasy^®^ Blood and Tissue Kit (QIAGEN, Hilden, Germany). The DNA sample was stored at −20 °C in ddH_2_O. This DNA was used as a template to amplify the 16S rRNA gene with primers 27f (5′-AGAGTTTGATCCTGGCTCAG-3′) and 1522r (5′-AAGGAGGTGATCCAGCCGCA-3′) [29]. Each reaction mixture (final volume, 50 μL) consisted of 20 mM Tris-HCl (pH 8.4), 3 mM MgCl_2_, each deoxynucleoside triphosphate at a concentration of 0.2 mM, each primer at a concentration of 0.2 μM, 1.25 U of *Taq* polymerase, and 1 μL of appropriately-diluted template DNA. The PCR program comprised of: predenaturation at 94 °C for 5 min; 35 cycles of denaturation 94 °C for 30 s, annealing at 56 °C for 20 s, extension at 72 °C for 40 s, and a final extension at 72 °C for 10 min. The sequences representing the YC152 bacterium were compared with the NCBI database by using BLASTN, and the closest match to the bacterial isolate was retrieved.

### 2.2. Factors Affecting the Cr(VI) Removal Efficiency of Isolate YC152

To obtain the growth curve of the isolate YC152, YC152 was cultured in 300-mL LB broth supplemented with 0.25 mM Na_2_Cr_2_O_7_ (26 mg/L of Cr(VI)) and incubated at 35 °C at pH 7.0 under anaerobic conditions. Samples were obtained every 2 h during the culture period. Each sample was immediately measured at 600 nm by using a UV-VIS spectrophotometer. At the same time, cell numbers in samples were determined using the plate count method after 24 h of cultivation at 35 °C.

To determine the effects of different environmental factors on the Cr(VI) removal efficiency of the isolate YC152, different pH values (5–10), culture temperatures (20–40 °C), and NaCl concentrations (5–25 g/L), and four simulated wastewater types containing Cr(VI) were tested. Simulated Wastewater I contained diluted LBCr medium (1/1000 LB supplemented with 26 mg/L of Cr(VI)). Simulated Wastewater II contained diluted LBCr medium, 15 mg/L Cu^2+^, 25 mg/L Zn^2+^, 10 mg/L Ni^2+^, 5 mg/L Na^+^, and 5 mg/L SO_4_^2−^. Simulated Wastewater III contained diluted LBCr medium, 30 mg/L Cu^2+^, 50 mg/L Zn^2+^, 20 mg/L Ni^2+^, 10 mg/L Na^+^, and 10 mg/L SO_4_^2−^. Simulated Wastewater IV contained diluted LBCr medium, 60 mg/L Cu^2+^, 100 mg/L Zn^2+^, 40 mg/L Ni^2+^, 20 mg/L Na^+^, and 20 mg/L SO_4_^2−^. In these batch experiments, the diluted LBCr medium was used. The shaker culture was maintained at 35 °C and run at 200 rpm, and the cell number of inoculated YC152 was 2.0 × 10^8^ cfu/mL, unless stated otherwise. The Cr(VI) removal efficiency of the isolate was analyzed after 48-h cultivation periods. All of the experiments were conducted at least in triplicate.

### 2.3. MFC Construction

The structure of a MFC biosensor was a typical two-chamber unit. The rectangular anode and cathode compartments were constructed from polyacrylic plastic (working volume: 170 mL each) and physically separated using a proton exchange membrane (PEM; Nafion 117, DuPont Co., Fayetteville, NC, USA) with a surface area of 49 cm^2^. The plain porous carbon paper (30.25 cm^2^ surface area) was used as electrodes with an OK line or wire connecting them through a variable resistor. Four pores were located at the top of the MFC for in and out of the electrode wire, addition and sampling of solutions, and online detection of ORP and pH. The anolyte of the MFC contained the diluted LBCr medium, unless stated otherwise, and the catholyte of the MFC contained 50 mM phosphate buffer (pH 7) and 100 mM NaCl solutions. The anode compartment was maintained anoxic by purging with nitrogen gas.

### 2.4. Cell Immobilization and Biosensor Operation

The anode compartment of the MFC biosensor with a 500-Ω resistor was continuously introduced with the LB medium containing the isolate YC152 (2.0 × 10^8^ cfu/mL) for cell immobilization at 10-d LRT. The feed solution and anode compartment were kept anoxic by purging with nitrogen gas. When the voltage of the MFC reached a steady state (approximately 572 mV) after 20-d operation, the biofilm in the anode of the MFC was considered stable or mature. Thus, the effects of operation parameters on MFC characteristics were evaluated.

The 1/1000 LB medium was used as the anolyte to evaluate the performance of the MFC biosensor. The circuit was adjusted using variable resistance (50–3900 Ω) to obtain a polarization curve of the MFC. Subsequently, one ml of Cr(VI) with a final concentration (0.0125–5 mg/L) was added to the 1/1000 LB medium (original anolyte) to establish the relationship between the Cr(VI) concentration and voltage output of the MFC biosensor. To examine the stability of the MFC, 2/3 of the 1/1000 LB medium in the MFC was replaced by a fresh medium when the voltage decreased to approximately 1/10 of the maximal value over a 27-d period. The MFC biosensor was operated in a batch mode for at least 12 h for each operation parameter to obtain a stable voltage production. The MFC biosensor was placed in a temperature-controlled chamber maintained at 35 °C.

### 2.5. Cr(VI) Measurement in Artificial and Real Wastewater

Cr(VI) concentrations in artificial and real water samples (including drinking water, groundwater, domestic wastewater, and electroplating wastewater) were measured using the MFC biosensor, modified AAS technique, or/and colorimetric method. To evaluate the feasibility of the MFC biosensor, the original anolyte in the MFC was completely replaced by artificial and real water samples. Artificial wastewater contained 1/1000 of the LB medium supplemented with different Cr(VI) concentrations (0.05–3.5 mg/L). Furthermore, 169 mL of real wastewater was supplemented with 1 mL of the 17/100 LB medium to maintain the LB concentration in the anolyte of the MFC. According to the relationship between the Cr(VI) concentration and voltage output of the MFC biosensor (described in Section 2.4), the Cr(VI) concentration in the water sample was easily obtained. In this study, the reaction time was set at 45 min. All experiments were conducted using five separate MFCs, and all analyses were conducted in triplicate.

### 2.6. Analysis

Na_2_Cr_2_O_7_ of special grade chemicals was dried at 200 °C for 1 h and left in a desiccator. Subsequently, 25.2 mg of Na_2_Cr_2_O_7_ was weighed and dissolved in water and diluted to 100 mL. The diluted solution was used as a standard stock solution of 100 mg/L of Cr (VI). The Cr(VI) was first chelated with ammonium pyrrolidine dithiocarbamate (APDC) and then extracted with methyl isobutyl ketone (MIBK) [30]. The extract was aspirated into the flame of the atomic absorption spectrophotometer (Hitachi, Tokyo, Japan). The colorimetric method for Cr(VI) measurement was performed as described previously [31].

The MFC potential or voltage was measured using a multimeter (Model 2700, Keithley Instruments, Inc., Solon, OH, USA). Data were digitally recorded every minute on a computer by using an interface card (Model PCI-488, Keithley Instruments, Inc.). The measured voltage (*V*) was converted to current (*I*) according to the following relationship: voltage (*V*, *volt*) = current (*I*, *amp*) × resistance (*R*, *ohm*). The power (*P*, *watt*) was calculated as *P* = *I* × *V* and then normalized using the surface area of the anode. All experiments were conducted using five separate MFCs, and all analyses were conducted at least in triplicate.

## 3. Results and Discussion

### 3.1. Identification and Characterization of Isolate YC152

The 16S rRNA of the isolate YC152 exhibited the highest sequence similarity (97.8%) to the 16S rRNA of *O. anthropi*. The strain was Gram-negative, rod-shaped, facultatively anaerobic, and motile by means of peritrichous flagella. After growth on nutrient agar for 24 h, the colonies had an average diameter of 1.2 μm and were circular, smooth, low convex, and non-pigmented. *O. anthropi* YC152 belonged to the α subclass of Proteobacteria.

The growth curve of *O. anthropi* YC152 exhibited the lag phase in the first 6 h, followed by the log phase from 10 to 22 h, and then entered the stationary phase under the anaerobic condition. According to the growth curve, the inoculation time was set at 20 h for further experiments. In addition, the OD_600_ value of cell growth was proportional to the log cell number, and the regression equation was determined to be *y* (log cell number) = 2.26*x* (OD_600_) + 4.263 (*r*^2^ = 0.991). We calculated the cell number of *O. anthropi* YC152 by using this equation. According to the growth curve, the specific growth rate was determined to be 0.482 h^−1^.

**Figure 1:**
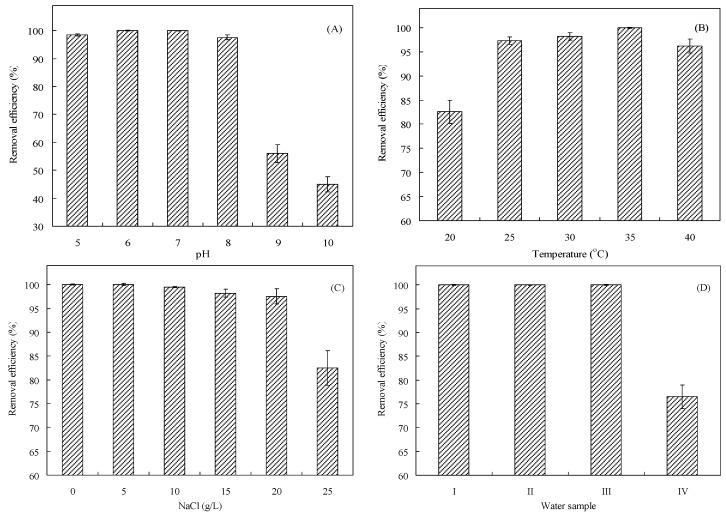
Effects of (**A**) pH (temperature: 30 °C, NaCl concentration: 0 M); (**B**) temperature (pH: 7, NaCl concentration: 0 M); (**C**) NaCl concentration (temperature: 30 °C, pH: 7); and (**D**) various water quality on the Cr(VI) removal efficiency of *O. anthropi* YC152 (Cr(VI) concentration was: 26 mg/L).

Since the pH of wastewater containing Cr(VI) might be weakly acidic, the effect of pH on the Cr(VI) removal efficiency of *O. anthropi* YC152 was evaluated. As presented in Figure 1A, the weak acidic condition (i.e., pH 5 and 6) did not affect the Cr(VI) removal efficiency of *O. anthropi* YC152 (98.5%–100%); however, the basic condition (i.e., pH 9 and 10) reduced the Cr(VI) removal efficiency to 45%–56%. Thus, the MFC biosensor was used for measuring the Cr(VI) concentration when the pH value of the solution was pH 5–8. This characteristic can favor the operation of the MFC biosensor for measuring the Cr(VI) concentration. Figure 1B illustrates the effect of temperature on the Cr(VI) removal efficiency of *O. anthropi* YC152. The results revealed that high Cr(VI) removal efficiencies were observed in the range of 25–40 °C, with the highest removal efficiency of 100% observed at 35 °C. However, the psychrophilic temperature (e.g., 20 °C) was not beneficial to the Cr(VI) removal efficiency of *O. anthropi* YC152. The optimal pH and temperature for Cr(VI) removal by using *O. anthropi* observed in this study were consistent with those reported by Sultan and Hasnain (2012) [32]. Electrolytes, such as Na^+^ and Cl^−^, exist at a high concentration in wastewater and can potentially affect the activity of microbes and the function of ion-exchange membranes in MFCs [23]. Thus, the effect of NaCl on the Cr(VI) removal efficiency of *O. anthropi* YC152 was investigated. The average Cl^−^ concentration in seawater is approximately 20 g/L [33]. As presented in Figure 1C, the Cr(VI) removal efficiency of *O. anthropi* YC152 was not significantly affected by increasing Cl^−^ concentrations (0–20 g/L); the removal efficiency was 97.5%–100%. In addition, the halotolerant characteristics have been reported by Wang et al. [34]. These results suggest that the MFC biosensor inoculated with *O. anthropi* YC152 has potential for measuring Cr(VI) concentrations in seawater. To evaluate the effect of coexisting ions on the Cr(VI) removal efficiency of *O. anthropi* YC152, different simulated wastewaters containing Cr(VI) were investigated. Figure 1D indicates that the Cr(VI) removal efficiency of *O. anthropi* YC152 was 100% when the wastewater contained 0–30 mg/L Cu^2+^, 0–50 mg/L Zn^2+^, 0–20 mg/L Ni^2+^, 0–10 mg/L Na^+^, and 5–10 mg/L SO_4_^2−^ (water samples I–III). However, the relatively low Cr(VI) removal efficiency (76.5%) of *O. anthropi* YC152 was observed when the wastewater contained 60 mg/L Cu^2+^, 100 mg/L Zn^2+^, 40 mg/L Ni^2+^, 20 mg/L Na^+^, and 20 mg/L SO_4_^2−^ (water samples IV). These results suggest that the MFC biosensor inoculated with *O. anthropi* YC152 has a high potential for Cr(VI) removal and measurement in different water bodies.

### 3.2. MFC Operation

**Figure 2:**
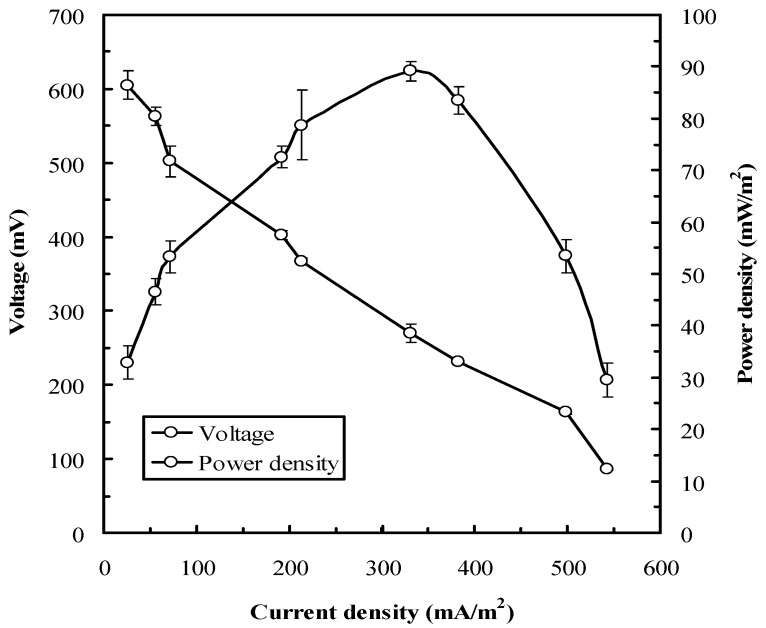
Polarization and power curves obtained from the MFC biosensor inoculated with *O. anthropi* YC152 during the stable phase of power generation (operating temperature: 35 °C, anolyte: 1/1000 LB, catholyte: 50 mM phosphate and 100 mM NaCl).

Since the outer layers of most bacteria are composed of nonconductive compounds that hinder the electron transfer to the anode, exoelectrogens in the anodic chamber of an MFC are crucial. Zuo et al. (2008) first reported the exoelectrogenic property of *Ochrobactrum* sp. and demonstrated its potential in MFC applications [35]. We observed that the voltage and power density of the MFC biosensor inoculated with *O. anthropi* YC152 were a function of the current density under various external resistances (50–3900 Ω). As presented in Figure 2, the MFC potential decreased as the current density increased, and the open circuit voltage was approximately 630 mV. Under such conditions, the maximum power density was 89.1 ± 1.2 mW/m^2^ and the optimal external resistance was 270 Ω. In addition, our results indicated that the decrease in MFC voltage had three different slopes because of the activation loss, ohmic loss, and mass transport loss. This result is in accordance with the characteristic of the MFC type [36]. Hence, the external resistance was set at 270 Ω for subsequent experiments.

**Figure 3:**
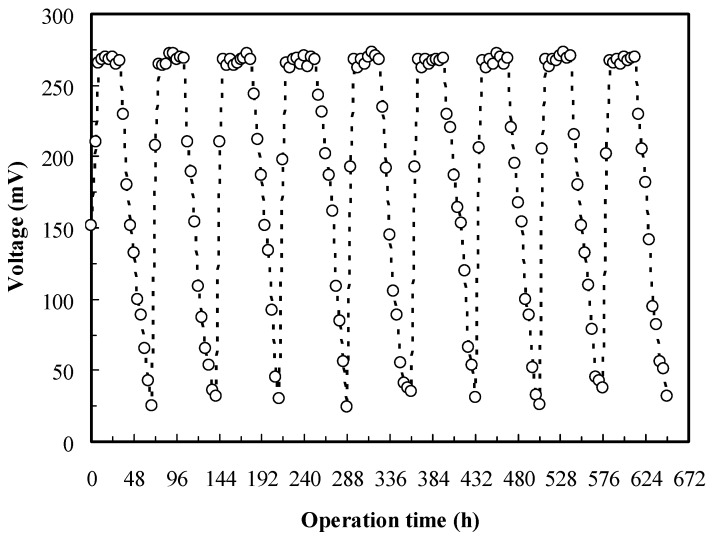
The stability of MFC biosensor inoculated with *O. anthropi* YC152 (operational temperature: 35 °C, resistance of external circuit: 270 Ω, anolyte: 1/1000 LB, catholyte: 50 mM phosphate and 25 mM NaCl).

The stable performance of the MFC biosensor during a desired operational period is essential for a reliable biosensor system. Figure 3 reveals that the complete voltage cycle (25–268–25 mV) was approximately 72 h for the *O. anthropi* YC152 MFC. When two-thirds of the anolyte was replaced with the fresh medium, the MFC reached the stable maximum voltage (268.0 ± 2.8 mV) in 8 h and continued until 32 h. The results of nine similar cycles in the 27-d operation demonstrated the operational stability of the MFC biosensor. Theoretically, when the anolyte in the MFC was periodically refreshed, the MFC biosensor could be continuously maintained.

### 3.3. Relationship between Cr(VI) Concentration and Voltage Output

Under optimal operation conditions, the relationship between the Cr(VI) concentration and voltage output of the MFC biosensor was determined. Since the MFC was designed to function as a Cr(VI) warning device to conform with water quality regulations, the 1/1000 LB medium supplemented with different Cr(VI) final concentrations (0.0125–5 mg/L) was introduced into the anode compartment of the MFC biosensor in a batch mode to establish their relationships. In the anode compartment of the MFC, Cr(VI) acted as an electron acceptor; thus, the MFC voltage was expected to decrease with an increasing Cr(VI) concentration. The results revealed that the MFC voltage decreased with reaction time. Higher Cr(VI) concentrations required longer reaction times to achieve a stable voltage output. However, only 15–45 min of reaction time was required for various Cr(VI) concentrations for stable voltage production. Moreover, compared with the original anolyte without Cr(VI), the anolyte supplemented with 1 mg/L of Cr(VI) significantly reduced the MFC voltage by 45%. Compared with the extent of voltage decrease in the cube MFC developed by Liu et al. (2014) and the flat microliter membrane-based MFC developed by Xu et al. (2015), the Cr(VI) MFC biosensor was more sensitive [37,38]. The signal amplification in our system would facilitate measuring the Cr(VI) concentration in water samplers. In this study, a stable voltage production in the MFC was observed in 15–45 min when different Cr(VI) concentrations (0.0125–5 mg/L) were introduced. The 5–15 min of recovery time of this biosensor was required in accordance with the Cr(VI) concentrations.

**Figure 4:**
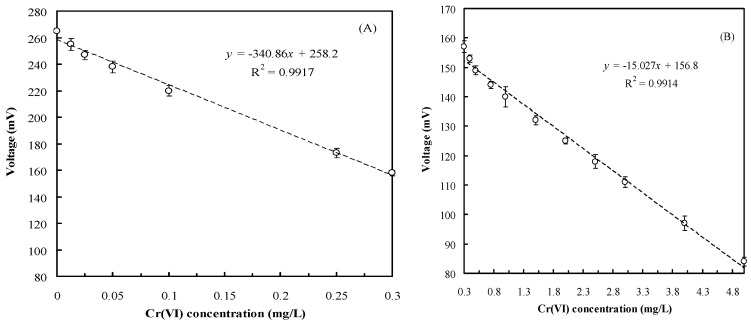
Relationship between Cr(VI) concentration and voltage output of the MFC biosensor: (**A**) Cr(VI) concentration: 0.0125 to 0.3 mg/L; (**B**) Cr(VI) concentration: 0.3 to 5 mg/L (operational temperature: 35 °C, resistance of external circuit: 270 Ω, anolyte: 1/1000 LB supplemented with different Cr(VI) concentration, catholyte: 50 mM phosphate and 25 mM NaCl, response time: 45 min).

Figure 4 presents the relationship between the Cr(VI) concentration and voltage output of the MFC biosensor. Two satisfactory linear relationships were observed between the Cr(VI) concentration and voltage output for various Cr(VI) concentration ranges. Figure 4A indicates that the regression equation for the Cr(VI) concentration and the voltage output of the MFC biosensor was determined to be *y* = −340.86*x* + 258.2 (*r*^2^ = 0.9917) when Cr(VI) concentrations ranged from 0.0125 to 0.3 mg/L. Furthermore, Figure 4B indicates that the other regression equation was determined to be *y* = −15.027*x* + 156.8 (*r*^2^ = 0.9914) when the Cr(VI) concentration ranged from 0.3 to 5 mg/L. The different relationships observed in different Cr(VI) concentrations may be because of Cr(VI) reduction rates and bacterial characteristics [32]. Hence, on the basis of these two equations, the Cr(VI) concentration in the water samples can be rapidly determined using the developed MFC biosensor. To prevent the MFC voltage decreasing because of the toxicity of Cr(VI) to microbes, the cell numbers of *O. anthropi* YC152 were evaluated by using the plate count method after experiments. The results revealed that the changes in the cell numbers (2.8–6.5 × 10^7^ cfu/mL) after the addition of different Cr(VI) concentrations were not significant.

### 3.4. Cr(VI) Measurement in Artificial and Real Wastewater

To evaluate the feasibility of using the MFC biosensor to measure Cr(VI) concentrations in wastewater, different artificial wastewaters containing Cr(VI) were synthesized, and the Cr(VI) concentrations of the samples were measured using the MFC biosensor and through AAS (Table 1). As listed in Table 1, compared with values determined using the MFC biosensor, those determined through AAS were slightly more accurate. Furthermore, compared with the standard Cr(VI) concentration, the deviation of the MFC biosensor was less than 10% when the Cr(VI) concentration in water samples was <0.5 mg/L. The precise MFC biosensor can be used as an early warning device for determining Cr(VI) effluents. Moreover, when values determined using the MFC biosensor and AAS were compared, <10% deviation (−3.8% to 9.8%) was observed for all tested concentrations. These results indicate that the accuracies of the developed Cr(VI) MFC biosensor and the standard test method employing AAS are similar for measuring the Cr(VI) concentration, ranging from 0 to 3.5 mg/L.

Different water samples usually contain different types and concentrations of organic compounds, metal ions, and pH. Thus, the possible effects of these coexisting compounds on the Cr(VI) concentration measured using the MFC biosensor were evaluated. Table 2 lists Cr(VI) concentrations in various real water samples measured through a modified atomic absorption spectroscopy technique, by using the MFC biosensor, or using colorimetric method. Compared with Cr(VI) concentrations measured through AAS, those measured using the MFC biosensor in drinking water, groundwater, and electroplating wastewater were more accurate and had low deviations (−7.7% to 9.2%). Furthermore, Cr(VI) concentrations in domestic wastewater measured using the MFC biosensor exhibited higher deviations (−17.7% to −18.4%) than did those measured through AAS. Similar results were also found when those measurements obtained by the MFC biosensor were compared with Cr(VI) concentrations measured by using colorimetric method. These results seem to conflict with those in Table 1, which indicate that the deviation was <10% when Cr(VI) concentrations in water samples were <5 mg/L. The high adaptability of *O. anthropi* YC152 to pH, temperature, salinity, and water quality is presented in Figure 1. Thus, this inconsistency in the results may be attributed to the content of organic compounds in water samples. Since organic concentrations in domestic wastewater were higher than those in other tested waters or wastewaters, a high initial voltage would be produced because of the high electron donors (organic compounds), followed by a decrease in voltage. According to the estimated equation of the Cr(VI) concentration presented in Section 3.3, the Cr(VI) concentration would be underestimated. Compared with previous studies [9,13,15,17], our results demonstrate that the developed Cr(VI) biosensor has an appropriate detection limit range for Cr(VI) (0.0125–5 mg/L) and can measure the concentration in the short time of 45 min. Thus, the developed MFC biosensor has the potential to be used as an early warning device to protect ecosystems.

## 4. Conclusions

In this study, we developed an MFC biosensor inoculated with *O. anthropi* YC152 to rapidly determine Cr(VI) at trace concentrations. Our results revealed that the MFC biosensor can be used as a reliable warning device for Cr(VI) determination in different water samples with low deviation and high sensitivity, which had previously not been fully studied. The physiological characteristics of *O. anthropi* YC152, including facultative anaerobe, Cr(VI) reduction, exoelectrogen, and high adaptability to pH, temperature, salinity, and water quality, demonstrated that it was suitable for application in an MFC to determine Cr(VI). The developed MFC biosensor can, in only 45 min, evaluate whether Cr(VI) concentrations in water samples conform with the effluent regulations and the maximum allowable concentration in water. Thus, the application of the MFC biosensor as an early warning device for Cr(VI) determination is promising.

## Figures and Tables

**Table 1:** Cr(VI) measurement from artificial wastewater by atomic absorption spectroscopy and MFC biosensor.

	Standard Cr(VI) Concentration of (mg/L)					
	0.05	0.1	0.25	0.5	1.5	3.5
AAS ^1^	0.051 ± 0.01	0.112 ± 0.01	0.25 ± 0.02	0.53 ± 0.03	1.58 ± 0.02	3.46 ± 0.05
MFC biosensor	0.053 ± 0.01	0.109 ± 0.02	0.26 ± 0.06	0.51 ± 0.02	1.72 ± 0.01	3.80 ± 0.03
Deviation (%) ^2^	2	12	0	6	5.3	−1.1
Deviation (%) ^3^	6	9	4	2	14.7	8.6
Deviation (%) ^4^	3.9	−1.8	4	−3.8	8.9	9.8

^1^ Modified atomic absorption spectroscopy technique; ^2^ The determined value by atomic absorption spectroscopy compared to standard Cr(VI) concentration; ^3^ The determined value by MFC biosensor compared to standard Cr(VI) concentration; ^4^ The determined value by MFC biosensor compared to that by modified atomic absorption spectroscopy technique.

**Table 2:** Cr(VI) measurement from real wastewater by atomic absorption spectroscopy, MFC biosensor, and colorimetric method.

	Drinking Water		Groundwater		Domestic Wastewater		Electroplating Wastewater	
	A	B	A	B	A	B	A	B
AAS ^1^	0.015 ± 0.001	0.036 ± 0.002	0.052 ± 0.009	0.120 ± 0.018	0.49 ± 0.031	0.62 ± 0.052	2.06 ± 0.082	4.31 ± 0.069
MFC biosensor	0.016 ± 0.002	0.038 ± 0.003	0.048 ± 0.010	0.131 ± 0.015	0.40 ± 0.026	0.51 ± 0.026	2.19 ± 0.051	4.65 ± 0.031
Colorimetric method	0.017 ± 0.005	0.037 ± 0.005	0.050 ± 0.012	0.128 ± 0.026	0.46 ± 0.051	0.58 ± 0.041	2.13 ± 0.062	4.38 ± 0.064
Deviation (%) ^2^	6.7	5.6	−7.7	9.2	−18.4	−17.7	6.3	7.9
Deviation (%) ^3^	−5.9	2.7	−4.0	2.3	−13.0	−12.1	2.8	6.2

^1^ modified atomic absorption spectroscopy technique; ^2^ compared to the value measuring by AAS; ^3^ compared to the value measuring by colorimetric method.
